# The Management of Postoperative Atrial Fibrillation (POAF): A Systematic Review

**DOI:** 10.7759/cureus.42880

**Published:** 2023-08-03

**Authors:** Dakshin Meenashi Sundaram, Advait M Vasavada, Chetna Ravindra, Vinayak Rengan, Pravin Meenashi Sundaram

**Affiliations:** 1 Internal Medicine, ESIC Medical College and PGIMSR, Chennai, IND; 2 Internal Medicine, M P Shah Medical College, Jamnagar, IND; 3 General Surgery, Madras Medical College, Chennai, IND; 4 Pediatric Surgery, SMS Medical College, Chennai, IND; 5 General Surgery, Apollo Specialty Hospital, Chennai, IND

**Keywords:** new onset atrial fibrillation, af rhythm control, af rate control, post-operative atrial fibrillation, post operative arrhythmia, atrial fibrillation management

## Abstract

Postoperative atrial fibrillation (POAF) refers to new-onset atrial fibrillation (AF) that develops after surgery and is associated with an increased risk of mortality and thromboembolic events. The optimal management and treatment methods for POAF complications are not yet fully established. This systematic review aimed to evaluate the various treatment and management approaches currently available in terms of their suitability, efficacy, and side effects in handling POAF incidence post-surgery.

Google Scholar and PubMed electronic databases were searched extensively for relevant articles examining the various management techniques currently used to manage POAF and published between 2018 and 2023. Data were collected on the type of surgery the patients underwent, POAF definition period, intervention, and outcome of interest. Following a systematic assessment guided by the inclusion criteria, 10 of the 579 studies retrieved were included in this study, and 293,417 POAF cases were recorded. Three of these studies used different rhythm control and rate control treatments to manage POAF cases, while seven studies used various anticoagulation therapies to manage POAF incidence. For asymptomatic patients within one to three days of surgery, rate control is sufficient to manage POAF, and routine rhythm control is not needed; rhythm control should be reserved for patients who develop complications such as hemodynamic instability. Anticoagulation was performed in patients whose POAF exceeded four days after surgery. Anticoagulation was associated with an increased risk of mortality, stroke, thromboembolic events, and major bleeding in patients who underwent coronary artery bypass graft (CABG) surgery. In contrast, in a few other studies, anticoagulation treatment led to improved outcomes in patients who developed POAF. A wide range of management methods are available for POAF after different types of surgery. However, there is only limited evidence to guide the clinical practice. The data available are mainly retrospective and insufficient to accurately evaluate the efficacy of the various management methods available for POAF. Future research should make efforts to standardize the treatment for this condition.

## Introduction and background

Postoperative atrial fibrillation (POAF) is defined as new-onset atrial fibrillation (AF) occurring immediately after surgery and is the most common type of secondary AF (AF attributed to identifiable, primary, and acute conditions) [1]. POAF is the most prevalent and potentially fatal cardiac complication occurring postoperatively. POAF affects approximately 25-55% of cardiac surgery patients [2]. In approximately 90% of cases, patients develop POAF within the first six days following the operation, coinciding with the peak of the systemic inflammation response after surgery [3]. POAF is an indicator of both short-term and long-term cardiovascular problems, such as stroke, infarction, thromboembolism, and cardiac arrest, and may require reoperation owing to internal bleeding [4,5]. In addition to spending an average of 3.7 more days in the hospital, POAF patients have a two-fold greater risk of all-cause 30-day and six-month mortality [6,7].

POAF is distinguishable from other forms of AF since it is characterized by a unique presentation compared to other forms of AF. POAF typically arises in patients within the first six days post-surgery and then returns to a normal sinus rhythm. Although the definitive mechanism behind POAF is still not fully understood, etiologies currently associated with POAF usually require a trigger as well as a vulnerable atrial substrate change, normally originating from the pulmonary veins and other atrial areas [8]. Moreover, numerous biochemical events that cause metabolic derangements in cardiomyocytes affect electrophysiological, contractile, and structural cellular characteristics in AF pathogenesis [9,10]. Medical management or treatment is usually based on the understanding of etiology, and given that the mechanism of the pathology of POAF is still elusive, the scope for successful preventative and treatment measures for POAF is limited. Recent developments and research have shown that POAF is partly preventable [11]. However, scarce and controversial data, lack of knowledge on independent risk factors, and effective interventions coupled with inconsistent clinical databases have limited the progress of any milestone advancement in the prevention of POAF. Despite the continuous and rapid growth of the POAF-related literature, the prevalence of POAF has remained constant over the past 30 years. Several factors such as myocardial infarction (MI), hypertension, heart failure, atrial fibrosis, heart disease, male sex, obesity, and a history of arrhythmias have been associated with a higher risk of developing POAF [11]. This systematic review provides a comprehensive and updated summary of the therapeutic management and treatment procedures that are currently available for POAF, including an analysis of the efficiency and side effects of these strategies.

## Review

Material and methods

This systematic literature review was conducted in accordance with the Preferred Reporting Items for Systematic Reviews and Meta-Analyses (PRISMA) guidelines. For the bibliographies, a systematic search was exhaustively performed on two electronic databases (PubMed and Google Scholar) to retrieve studies that evaluated the management of POAF. The systematic review protocol was registered in PROSPERO (International Prospective Register of Systematic Reviews) CRD42022369158.

Search Strategy

A comprehensive and systematic computerized search was conducted using a set of keywords or phrases in addition to Boolean expressions “OR” and “AND.” The keywords and phrases used to navigate the databases were as follows: (“management” OR “treatment” OR “therapeutic measures”) AND “postoperative atrial fibrillation” OR “POAF” OR “PAF” or “atrial fibrillation after surgery”). The search was customized to retrieve studies published between January 2018 and February 2023.

Eligibility Criteria

Three independent reviewers applied the following inclusion criteria to select studies for inclusion in this review:

· Primary and secondary studies evaluating the treatment or management of POAF.

· Studies published from January 2018 to February 2023 in the English language.

· Clinical trials, controlled clinical trials, meta-analyses, multicenter studies, observational studies, randomized controlled trials (RCTs), and systematic reviews.

Studies were excluded based on the following exclusion criteria:

· Studies that used non-human populations.

· Studies published before 2018, to ensure that only up-to-date information was used.

· Case series and case reports.

Data Extraction

Two independent investigators were tasked with performing data extraction, adhering to the predefined eligibility features of the studies and the PICO (population, intervention, control, and outcomes) guidelines. The extracted data included author information, population (size and characteristics), case intervention, and outcome of interest in POAF management.

Data Synthesis and Quality Appraisal

Applying a thematic approach, the two reviewers synthesized and screened the results of the publications included, thereby making it possible to analyze the results of all studies considered in this review. The reviewers independently inspected the abstracts and titles. The full texts of related studies were retrieved and reviewed. Relevant data were extracted from the studies, and the risk of bias was assessed using appropriate quality assessment tools. Consensus was sought through debate with a third author to resolve discrepancies, if necessary.

Ethical Approval

This systematic review required no ethical approval since it was a completely literature-based analysis of full-text primary and secondary published studies.

Results

Search Results

**Figure 1:**
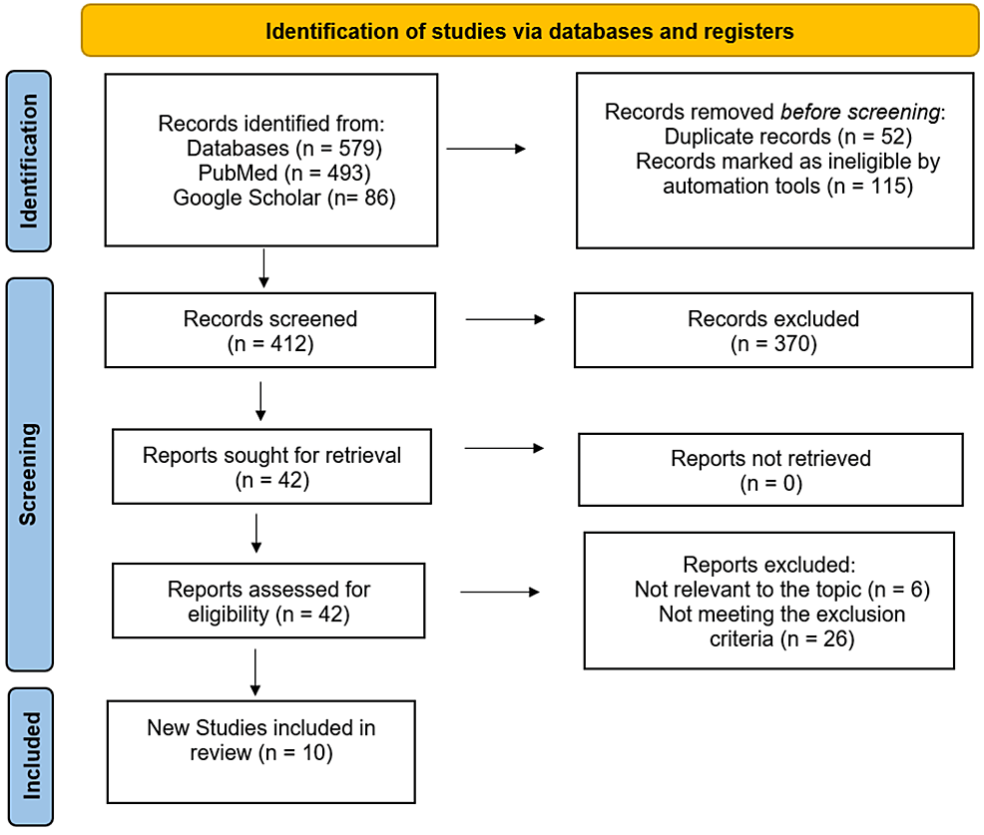
PRISMA flow chart depicting the selection of studies PRISMA: Preferred Reporting Items for Systematic Reviews and Meta-Analyses

The electronic search of the two databases yielded 579 articles, out of which 115 studies and another 52 were eliminated for unrelated titles and abstracts. After careful screening of the remaining 412 articles, 370 were excluded since they failed to meet the inclusion criteria. The 42 retrieved studies were assessed for eligibility. Among these, six did not discuss POAF management or treatment, and 26 were published before 2018 and were thus excluded. This review was based on the remaining 10 studies that fully met the inclusion criteria. The PRISMA flow chart for study selection is illustrated below in Figure 1 [12].

Study Characteristics

The characteristics of all the studies included in the review are summarized in Tables 1-2.

**Table 1:** Characteristics of primary studies POAF: postoperative atrial fibrillation; CABG: coronary artery bypass grafting; IS: ischemic stroke; TIA: transient ischemic attack; TEE: thromboembolic event; HF: heart failure; MB: major bleeding; PE: pulmonary embolism; OACs: oral anticoagulants; POD: postoperative days; RaC: rate control strategy (β-blockers/non-dihydropyridine calcium channel blockers); RhC: rhythm control strategy [anti-arrhythmic (P1)/DC cardioversion anticoagulation and amiodarone management]; CS: cardiac surgery; MI: myocardial infarction; CVS: cardiac valve surgery

Study ID		Population			Intervention	POAF			Outcomes	
Author, year	Study design	Sample size	Age, years	Sex, M/F	Management/treatment	POAF definition	Surgery undergone	Follow-up	Outcome measures	Main results
Bruggmann et al., 2021 [13]	Observational retrospective study	97 patients	Mean ±SD: 68.5 ±9.6	82/15	RaC RhC	1–3 POD	CS, CABG	-	Recurrence and duration of POAF. First POAF episode and return to sinus rhythm	There are not any definitive rules for managing POAF, which highlights the need for a bigger picture
Kalava and Pribish, 2018 [14]	Retrospective study	17,039 cesarean deliveries	Median (range): 33 (26–41)	0/17,039	RaC RhC	First 48 hours following cesarean section	Cesarean section	-	-	POAF is an uncommon complication that affects women who have C-sections and increases hospital stays and resource use
Riad et al.,2020 [15]	Retrospective study	200 patients	Mean ±SD: 68 ±12	122/78	Anticoagulation (warfarin)	30 POD	CABG, CVS	1 year	IS	After CABG, anticoagulation use was minimal but increased following bioprosthetic valve surgery
Riad et al., 2022 [16]	Retrospective propensity-matched analyses	38,936 patients	Median (range): 73 (69–77)	30,099/8837	Anticoagulation	-	CABG	30 days, 30–180 days, and 180 days	Higher short-long term mortality. Higher readmission rates. IS, TIA, MB, and MI	With the development of new POAF after CABG, anticoagulation is linked to higher mortality and no decreased IS
Taha et al., 2021 [17]	Observational, longitudinal cohort study	POAF=7368. No POAF=17,155	Mean ±SD: POAF=70 ±8.0, No POAF=66 ±9.3	19,694/4829	Early-initiated OACs	Any new-onset AF during the first 30 POD	CABG	Median (range) in years: 4.5 (0–9)	Mortality (10.6%) IS (6.3%) TIA (3%) TEE (9.7%) HF (6.1%) PE (1%) MB (7.4%)	Early OAC in POAF was linked to increased MB risk rather than any decreased mortality or TEE risks

**Table 2:** Characteristics of included reviews HVS: heart valve surgery; ECG: electrocardiogram; CCM: continuous cardiac monitoring; ICD: International Classification of Diseases; DOACs: direct oral anticoagulants; NA: not applicable; MNE: major neurological events (stroke, TIA)

Author, year	Included studies (range)	Sample size	Surgery	Intervention (management)	POAF		Outcomes
					Definition	Detection	
Aguilar et al., 2021 [18]	NA	NA	CS	RaC RhC anticoagulation	2–4 POD	-	The RaC technique is sufficient for the majority of asymptomatic POAF patients, and anticoagulation should be started if POAF develops 48 to 72 hours after surgery
Gudbjartsson et al., 2020 [19]	NA	NA	CS	Sotalol and amiodarone	-	-	A thorough analysis of the pathogenesis of POAF during cardiac surgery, clinical risk factors, procedural risk factors, preventative strategies, and treatment are described
Koh et al., 2022 [20]	12 (2003–2022)	16,902 patients	CS	8587 DOACs; 8315 warfarin	-	-	Incidences of MNE and postoperative hemorrhage with DOACs were 7.3% (95% CI: 3.4–14.7%) and 2.2% (95% CI: 0.9–4.9%), respectively
Neves et al., 2022 [21]	10 (2009–2021)	203,946 patients	CABG, CS	Anticoagulation	-	ICD	Patients with POAF who underwent cardiac surgery were more likely to benefit from oral anticoagulation than those who underwent noncardiac surgery
Yao et al., 2021 [22]	17 (2000–2019)	44,908 adult patients of which 8929 POAF	CABG, HVS	Warfarin NOACs. Concomitant antiplatelet therapy	30–40 hrs post-surgery	ECG, CCM, ICD	While patients who were discharged on warfarin had a lower death rate, OAC use was linked to a lower risk of thromboembolic events

Quality Assessment

Two investigators independently evaluated the risk of bias of the included studies using the Newcastle-Ottawa Quality Assessment Scale for case-control studies and cohort studies, GRADE (Grading of Recommendations Assessment, Development, and Evaluation) certainty rating, and Assessment of Multiple Systematic Reviews 2 (AMSTAR 2) checklist for meta-analysis, which are depicted in Table 3, Table 4, and Table 5 respectively. All the studies were of low risk of bias with no effect on the outcomes of our results.

**Table 3:** Quality assessment of the included studies using the Newcastle-Ottawa Quality Assessment Scale

Study	Selection	Comparability	Outcomes	Rating
Bruggmann et al., 2021 [13]	2	1	2	Moderate
Kalava and Pribish, 2018 [14]	3	1	2	Moderate
Riad et al., 2020 [15]	2	1	2	Moderate
Riad et al., 2022 [16]	2	1	3	Moderate
Taha et al., 2021 [17]	2	1	3	Moderate

**Table 4:** Quality assessment of the included studies using the GRADE certainty rating GRADE: Grading of Recommendations Assessment, Development, and Evaluation

Study	GRADE certainty rating
Aguilar et al., 2021 [18]	High
Gudbjartsson et al., 2020 [19]	High

**Table 5:** Quality assessment of the included studies using the AMSTAR 2 grading AMSTAR 2: Assessment of Multiple Systematic Reviews 2

Study	AMSTAR 2 grade
Koh et al., 2022 [20]	Moderate
Neves et al., 2022 [21]	Moderate
Yao et al., 2021 [22]	Moderate

Discussion

This systematic review aims to evaluate the management approaches for POAF following different surgical procedures. POAF is typically transient and often requires no intervention or treatment. However, therapeutic management is required in high-risk patients with abnormal cardiac function, cerebral thromboembolism, or POAF duration >48 hours. The immediate postoperative setting presents unique challenges for the management of patients with POAF. Our findings showed that several methods have been used for the treatment and management of POAF. Approaches for the treatment of POAF include heart rate control, rhythm control, and antithrombotic therapy, which are the same approaches for treating chronic AF.

Our findings show that rhythm control strategies (such as antiarrhythmic, cardioversion, and amiodarone management) and rate control strategies (including β-blockers/non-dihydropyridine calcium channel blockers) are used to manage short-term complications when POAF is defined in a patient within the first 48 or 72 hours (or within three days) post-surgery (P1) [13,14,18]. Rhythm control and rate control are two broad therapies used for treating patients with POAF. Rhythm control is a strategy that focuses on returning to normal sinus rhythm and maintaining it using anti-arrhythmic drugs [23]. In contrast, rate control applies a single medication or a combination of negatively chronotropic medications to control heart rate. Rhythmic control methods for POAF have been favored to reduce the risk of systematic anticoagulation, promote faster recovery of full operation capacity, and reduce the duration of hospital stay and costs of hospitalization [23]. This study further illustrates that the rate control technique is sufficient for most asymptomatic patients with POAF.

Another study also reached the same conclusions, arguing that for asymptomatic patients within one to three days of surgery, rate control is sufficient to manage POAF, and that routine rhythm control is not needed; rhythm control should be used for patients who develop complications, such as hemodynamic instability [24]. Rate control techniques include β-blockers and antiarrhythmic drugs such as amiodarone and sotalol. Beta-1 receptor antagonists or blockers are inhibitors of β1-adrenergic receptors, which play a role in sympathetic stimulation in the myocardium. Increased sympathetic stimulation is one of the etiologies of POAF. β-blockers block these receptors, thereby reducing the stroke volume and cardiac output and decreasing the heart rate. β-blocker prophylaxis prior to cardiac surgery has been demonstrated as a highly effective POAF preventive strategy [24]. A Cochrane review and meta-analysis of 33 studies suggested that β-blockers resulted in a significant decrease in POAF incidence [odds ratio (OR): 0.33, 95% CI: 0.26-0.43) [5]. Cochrane analysis showed substantial heterogeneity among the studies (I^2^=55%). Notably, the Cochrane study might have overestimated β-blocker efficacy since the study relied on the discontinuation of background therapy with β-blockers with the potential to increase the POAF incidence due to control group withdrawal [5]. Despite this, a trial that did not warrant any control group withdrawal yielded consistent results and confirmed the high efficacy of β-blockers. However, β-blockers did not significantly affect mortality, stroke, or length of hospitalization [5]. In addition, a Cochrane systematic review of 63 randomized trials also inferred that β-blockers decreased the risk of POAF [with a relative risk (RR) of 0.50 (95% CI: 0.42-0.59; heterogeneity I^2^=59%] [25]. Although these studies agree that the use of β-blockers is an effective therapy for managing POAF after cardiac surgery, β-blockers are not commonly used for prophylaxis. This has been confirmed by our review, where the studies reviewed hardly reported β-blockers as an intervention for POAF. Another study carried out by the European Association of Cardiovascular Anesthetists (EACA) and the Society of Cardiovascular Anesthesiologists (SCA) reported alternative approaches to the perioperative use of β-antagonists [26].

The study also reported the use of amiodarone and sotalol [19] in the management of POAF after cardiac surgery. Other trials have proven that amiodarone is effective for POAF prevention [5] and has been granted Class IIA indication by European and American guidelines. Amiodarone is classified as an antiarrhythmic agent responsible for peripheral and coronary vasodilation. However, amiodarone poses serious risks such as increased liver enzymes, bradycardia, thyrotoxicosis, hyperthyroidism, and interstitial pneumonitis. These side effects may significantly outlast the discontinuation of use [27].

Seven studies in this review recorded the use of anticoagulation [15-18,20-22]. Two studies reported the use of warfarin [15,20]; one reported different types of anticoagulants, including direct oral anticoagulants (DOACs), warfarin, and concomitant antiplatelet therapy [22], while the remaining studies did not specify the type of anticoagulant used. With the development of new POAF after CABG surgery, DOACs have been associated with higher short-to long-term mortality, readmission, ischemic stroke, transient ischemic attack, major bleeding, and myocardial infarction [15-17,20-22]. A study by Taha et al. reported a mortality rate of 10.6%, ischemic rate of 6.3%, transient ischemic attack of 3%, thromboembolic events of 9.7%, heart failure of 6.1%, pulmonary embolism of 1%, and major bleeding of 7.4% after early initiation of OACs in 7368 patients who developed POAF after CABG [17]. Anticoagulation should be minimal after CABG but can be increased following bioprosthetic valve surgery.

In contrast, anticoagulation resulted in better outcomes in the treatment of POAF after non-CABG cardiac surgery. This outcome is consistent with other studies that have shown that anticoagulation reduces the risk of mortality in patients who develop POAF after non-CABG cardiac surgery but increases the risk of mortality, stroke, and major bleeding in those who develop POAF following noncardiac surgery [9,28]. This is a challenge because anticoagulation therapy presents two distinct patient groups, each with a high or low risk of AF recurrence. Therefore, a predictive framework should be developed to ensure that risks are eliminated in both these distinct populations.

This review further revealed that in cases where anticoagulation was used, POAF was defined beyond four days to 30 and 30 to 180 days post-surgery, and direct oral anticoagulation was preferred [15,16]. The duration of anticoagulation is critical because of the risk of early stroke or AF recurrence. Limited data are available to guide the timing and duration of anticoagulation therapy. However, studies show that timing should vary between 48 and 72 hours or earlier if there is a greater risk of thromboembolic events. The risk of gross complications and bleeding due to platelet dysfunction and cardiopulmonary bypass decreased in the early preoperative period. Other studies have shown that the risk of stroke and recurrent AF is highest within the first year of surgery, after which there is a steady decline [29,30]. Our review adds to the 2014 AATS guidelines for the prevention and management of perioperative atrial fibrillation and flutter for thoracic surgical procedures [11]. However, data on the long-term significance of POAF episodes are currently unavailable, and more research should be conducted to develop properly defined standard procedures for treating and managing POAF.

Limitations of the study

Despite our rigorous and comprehensive approach, this systematic review has some limitations that warrant consideration. Firstly, most of the studies were retrospective, and RCTs were scarce. Retrospective studies may be prone to sample bias, which may compromise the accuracy of the conclusions inferred. Moreover, the sample sizes in the studies reviewed were low, thus affecting our ability to assess outcomes and allow adjustments for covariables. Another limitation was the small number of articles that met our inclusion criteria, which means that we could not gather a substantial amount of evidence or data.

## Conclusions

There are several approaches that have been employed in the management of POAF after various types of surgeries. The different strategies or therapies for the treatment of POAF include rate control, rhythm control, and antithrombotic therapy. These techniques consist of a wide range of pharmacological and non-pharmacological treatments with variations in the duration of initiation, efficacy, and side effects. Our review contributes to the current guidelines for POAF; however, data on the long-term significance of POAF episodes are scarce, and further research should be conducted to develop properly defined standard procedures for treating and managing POAF.
